# Complete Mitogenome of *Oreolalax omeimontis* Reveals Phylogenetic Status and Novel Gene Arrangement of Archaeobatrachia

**DOI:** 10.3390/genes13112089

**Published:** 2022-11-10

**Authors:** Hongdi Luo, Lin Cui, Fuyao Han, Zhi He, Xiaolan Fan, Bo Zeng, Mingyao Yang, Deying Yang, Qingyong Ni, Yan Li, Yongfang Yao, Huailiang Xu, Jiandong Yang, Zhimin Wei, Tongqing Li, Dingqi Rao, Taiming Yan, Mingwang Zhang

**Affiliations:** 1 Key Laboratory of Livestock and Poultry Multi-omics, Ministry of Agriculture and Rural Affairs, College of Animal Science and Technology, Sichuan Agricultural University, Chengdu 611130, China; 2 Farm Animal Genetic Resources Exploration and Innovation Key Laboratory of Sichuan Province, Sichuan Agricultural University, Chengdu 611130, China; 3 College of Life Science, Sichuan Agricultural University, Ya’an 625014, China; 4 Institute of Millet Crops, Hebei Academy of Agriculture and Forestry Sciences, Shijiazhuang 050051, China; 5 Hebei Fisheries Technology Extension Center, Shijiazhuang 050051, China; 6 State Key Laboratory of Genetic Resources and Evolution, Kunming Institute of Zoology, Chinese Academy of Sciences, Kunming 650201, China

**Keywords:** *Oreolalax*, archaeobatrachia, mitogenome, phylogenetic analysis, gene rearrangement

## Abstract

Species of the genus *Oreolalax* displayed crucial morphological characteristics of vertebrates transitioning from aquatic to terrestrial habitats; thus, they can be regarded as a representative vertebrate genus for this landing phenomenon. But the present phylogenetic status of *Oreolalax omeimontis* has been controversial with morphological and molecular approaches, and specific gene rearrangements were discovered in all six published *Oreolalax* mitogenomes, which are rarely observed in Archaeobatrachia. Therefore, this study determined the complete mitogenome of *O*. *omeimontis* with the aim of identifying its precise phylogenetic position and novel gene arrangement in Archaeobatrachia. Phylogenetic analysis with Bayesian inference and maximum likelihood indicates *O*. *omeimontis* is a sister group to *O*. *lichuanensis*, which is consistent with previous phylogenetic analysis based on morphological characteristics, but contrasts with other studies using multiple gene fragments. Moreover, although the duplication of *trnM* occurred in all seven *Oreolalax* species, the translocation of *trnQ* and *trnM* occurred differently in *O*. *omeimontis* to the other six, and this unique rearrangement would happen after the speciation of *O. omeimontis*. In general, this study sheds new light on the phylogenetic relationships and gene rearrangements of Archaeobatrachia.

## 1. Introduction

The mitochondrial genome (mitogenome) is an important model system for studying molecular evolution, phylogeny, and genome structure [1,2]. Most vertebrate mitogenomes are characterized by several features shared with most eukaryote mitogenomes, such as a circular, double-stranded DNA molecules, which generally range from 15 to 20 kb [3]. This smaller organelle genome consists of 13 protein-coding genes (PCGs), two ribosomal RNA (rRNA) genes, 22 transfer RNA (tRNA) genes, and an AT-rich region, known as the control region (CR), which is involved in the initiation of transcription and replication of the mitogenome [4]. Due to their small size, cellular abundance, typical maternal inheritance, compact gene arrangement, and high rate of evolution, mitogenomes have been extensively used to test hypotheses about microevolution, to study population structure, phylogeny, and phylogeography at various taxonomic levels, and to identify cryptic species, which complements new developments using nuclear genes [1,5,6].

The genus *Oreolalax* (Anura, Megophryidae), which was reevaluated by Fei et al. [7] and has been accepted [8], currently consists of 19 species and is believed to originate from southwestern China [8] but has been found unexpectedly in Vietnam [9]. Wei et al. [10] considered that the genus *Oreolalax* contains the crucial morphological characteristics of species transitioning from aquatic to terrestrial habitats. Therefore, *Oreolalax* has become an important model for answering basic questions of morphology, development, and biogeographic evolution [11].

Six *Oreolalax* mitogenomes have been published [12,13,14,15,16] and have been shallowly analyzed for their phylogenetic relationships, which has led to a preliminary understanding of the phylogenetic relationships of *Oreolalax*. Furthermore, although gene rearrangement is rare in non-neobatrachians (i.e., archaeobatrachians) [17,18,19], specific gene rearrangements were discovered in all six *Oreolalax* mitogenomes. As for *O*. *omeimontis* (Omei toothed toad [20] or Omei lazy toad [21], common name), which can be distinguished from other *Oreolalax* species by its characteristic triangular markings between the eyes, this native Chinese species, mainly distributed in the Emei Mountains [22], somehow has only a few genetic fragments but no complete mitogenomic data. This further hinders the study of the evolution of gene rearrangements in the mitogenome of the genus *Oreolalax* and the Archaeobatrachia suborder.

The present phylogenetic status of *O. omeimontis* is still controversial and needs to be addressed, as morphology-based phylogenetic studies suggest that *O. omeimontis* and *O. lichuanensis* are sister branches [10], while phylogenetic studies based on molecular data (gene fragments) suggest that *O. omeimontis* was a sister to *O. multipunctatus* [9,23,24]. However, morphological characteristics change with the development of an individual, which poses methodological limitations [25], and differences in morphological characteristics may also be accompanied by variation in mitogenomes [26,27]. Meanwhile, previous research has also suggested that longer DNA sequences, such as a complete mitogenome, can provide more phylogenetic information than shorter gene fragments, which may more accurately reveal phylogenetic relationships and systematic taxonomic status [28,29,30]. Therefore, further research into the mitogenomic features, gene rearrangements and phylogenetics of *O*. *omeimontis* was carried out here to explore this poorly understood species and supplements the existing molecular data for *Oreolalax* taxa.

To fill the gap in our understanding of *O*. *omeimontis* and provide deep-level phylogenetic analysis for *Oreolalax*, this study sequenced the complete mitogenome of *O*. *omeimontis*, and described its characteristics and gene rearrangement. Moreover, we focused on the comparison between the seven mitogenomes of *Oreolalax* species to explore their features; maximum likelihood (ML) and Bayesian inference (BI) methods were employed to perform phylogenetic analyses to provide further information on the genus *Oreolalax*. This provides a valuable resource for further studies on the genetic diversity and phylogenetic analysis of *Oreolalax*.

## 2. Materials and Methods

### 2.1. Sample Collection and DNA Extraction

*O. omeimontis* specimens were collected from Wawushan National Forest Park, Sichuan, China in 2015. Tissue samples were preserved in 99% ethanol until DNA extraction. Total genomic DNA was extracted from muscle tissue according to protocols of the Ezup Column Tissue Genomic DNA Purification Kit (Sangon Biotech, Shanghai, China).

### 2.2. PCR Amplification and Sequencing

Twelve paired primers were used to amplify the complete mitogenome of *O. omeimontis* according to Zhang et al. [31]. PCR amplifications were performed with 2 μL of genomic DNA, 1 μL of each primer (10 μmol/L), 6 μL of ddH_2_O, 10 μL of 2×Taq Master-Mix Kit (Sangon Biotech, Shanghai, China). PCR reactions were performed under the following conditions: 5 min at 94 °C, followed by 35 cycles of 40 s at 94 °C, 40 s at 48 to 55 °C (depending on the primer combination), elongation at 72 °C for 1 to 3 min (depending on the fragment length), and a final extension at 72 °C for 10 min. PCR products were electrophoresed on a 1.0% agarose gel, then sequenced by Tsingke Biotechnology (Chengdu, China). DNA sequencing was performed on an ABI 3730 automatic DNA sequencer (Applied Biosystems, Foster City, CA, USA).

### 2.3. Sequence Assembly, Annotation, and Analysis

Each sequence was checked using the NCBI BLAST database (https://blast.ncbi.nlm.nih.gov/Blast.cgi, accessed on 10 May 2020), and then was assembled using both MEGA 7 [32] and SeqMan programs (Lasergene v7.1.0; DNAStar, Inc., Madison, WI, USA). A total of thirteen protein coding genes (PCGs) were first identified using the NCBI ORF Finder (https://www.ncbi.nlm.nih.gov/orffinder/, accessed on 10 May 2020) to specify the vertebrate mitochondrial genes. PCG, rRNA, and tRNA genes were annotated by the MITOS web server (http://mitos2.bioinf.uni-leipzig.de/index.py, accessed on 10 May 2020) [33] and tRNAscan-SE (http://trna.ucsc.edu/tRNAscan-SE/, accessed on 10 May 2020) [34] was used to compare and ensure the accuracy of the results; furthermore, both MITOS and tRNAscan-SE was used for recognition and prediction of tRNA gene secondary structure. The circular graphical map of the mitogenome was drawn using CGview web server (https://cgview.ca/, accessed on 10 May 2020) [35]. The relative synonymous codon usage (RSCU), start/stop codon, codon usages, and composition of nucleotides were analyzed in MEGA 7. AT-skew and GC-skew were calculated using the following formulas: AT-skew = (A - T) / (A + T) and GC-skew = (G - C) / (G + C) [36]. In addition, the same methods were used to reanalyze the published mitogenomes of six other *Oreolalax* species to supplement previously unanalyzed data.

### 2.4. Phylogenetic Analysis

To determine the phylogenetic position of *O. omeimontis*, 31 frogs (including *O. omeimontis* of this study) representing eight Anura families and three outgroup species (Table S1) were included for this analysis. The complete sequences of 13 PCGs of all species with deletions in the stop codons were aligned through MAFFT v7.313 [37] using default parameters. These 13 single-gene sequence alignments were concatenated using MEGA 7 and then used for downstream phylogenetic purposes. PartitionFinder v2.1.1 [38] was used to select the nucleotide substitution model of 13PCGs for the construction of the BI phylogenetic tree, and ModelFinder [39] was used to select for the ML phylogenetic tree, respectively. For ML and BI analysis, the nucleotides of 13 PCGs were codon partitioned and the best-fit model of each partition was selected (Table S2). Phylogenetic analysis was performed with the ML and Bayesian inference (BI) methods using IQ-TREE [40] and Mrbayes v.3.2.2 [41], respectively. For ML analysis, 1000 bootstrap replicates were used to calculate the bootstrap of the program. In the BI analysis, two independent runs for 10^7^ Markov chain Monte Carlo (MCMC) generations with four chains and sampling trees occurred every 1000 generations. The first 25% of trees were discarded as burn-in samples and the remaining trees were used to generate Bayesian consensus trees. The above software was running on the integrated and scalable desktop platform of PhyloSuite v1.1.16 [42]. The iTOL v4 website (https://itol.embl.de/, accessed on 10 May 2020) [43] was used to visualize and edit the results of both ML and BI trees.

## 3. Results and Discussion

### 3.1. Mitogenome Organization

**Figure 1:**
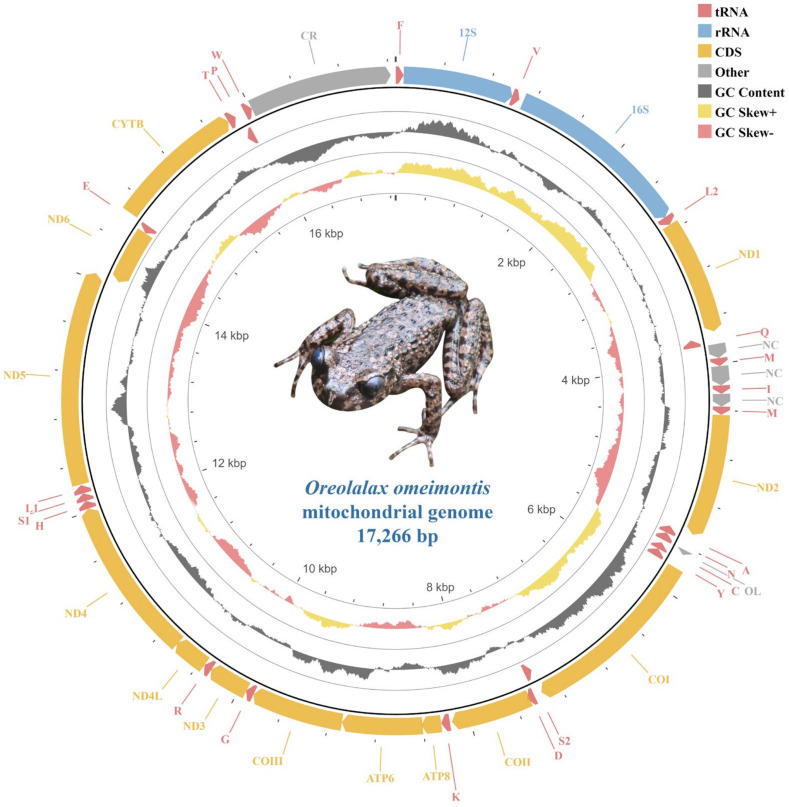
The mitochondrial genome of *Oreolalax omeimontis*. The direction of gene transcription is indicated by arrows. Arrows of different colors indicate corresponding gene types in the upper right of the figure.

The complete mitogenome of *O. omeimontis* was 17,266 bp in length (GenBank accession numbers: OP722573). The gene content was typical of other *Oreolalax* mitogenomes, which contained 13 PCGs, 2 rRNA genes, 23 tRNA genes (including an additional copy of *trnM*), a non-coding region known as the CR, and three intergenic regions (Figure 1). Eight tRNA genes and *nad6* were located on the L-strand (–), and the remaining genes (including the additional copy of *trnM*) were encoded by the H-strand (+) (Table 1).

The overall base composition of *O. omeimontis* was A: 27.6%, T: 31.3%, C: 26.2%, and G: 14.8%. The nucleotide composition of each part of the *O. omeimontis* mitogenome was A + T rich, ranging from 58.2% to 63.9% for the whole mitogenome of 13 PCGs, 2 rRNAs, 23 tRNAs and CR. The AT skewness (−0.063) and GC skewness (−0.278) values were both negative, indicating bias towards Ts rather than As, and bias towards Cs rather than Gs for the whole mitogenome of *O. omeimontis*. Table 2 shows the base composition of seven species of *Oreolalax* according to their mitogenomes, PCGs, tRNA genes, rRNA genes and control region. Compared to the six other *Oreolalax* species, *O. omeimontis* had the lowest A + T content in the complete mitogenome. Moreover, *O. omeimontis* had the lowest GC-skew values in tRNAs and PCGs, indicating that *O. omeimontis* had the lowest Gs content in tRNAs and PCGs out of the seven species of *Oreolalax*. In addition, the seven *Oreolalax* species’ AT-skew and GC-skew data had the same positive and negative values in tRNAs, PCGs, rRNAs, and CR, indicating that they were roughly the same in base biasing.

### 3.2. Protein-Coding Genes and Codon Usage

The total length of 13 PCGs for *O. omeimontis* was 11,389 bp. The coding regions ranged in size from 168 (*atp8*) to 1821 bp (*nad5*), accounting for approximately 66% of the entire mitogenome. Despite *nad6* being located on the L-strand (-), the remainder of the genes (*nad1*, *nad2*, *nad3*, *nad4l*, *nad4*, *cox1*, *cox2*, *cox3*, *atp8*, *atp6*, *nad5*, and *cob*) are all located on the H-strand (+). For the seven *Oreolalax* species, most 11 PCGs started with an ATN codon (ATG for *nad1*, *nad2*, *cox2*, *atp8*, *atp6*, *cox3*, *nad4l*, *nad4*, *nad5*, and *cob*; ATA for *nad3*) (Table S3). In contrast, most *cox1* and *nad6* started with the GTG codon. Ten PGCs ended with the stop codon TAA or TAG and included an incomplete stop codon T-- or TA-, while *cox1*, *nad5* (except for *O. jingdongensis*) and *nad6* terminated with the complete stop codon AGG or AGA (Table S3). These were all canonical mitochondrial initiation and termination codons for vertebrate mitogenomes [44,45].

**Figure 2:**
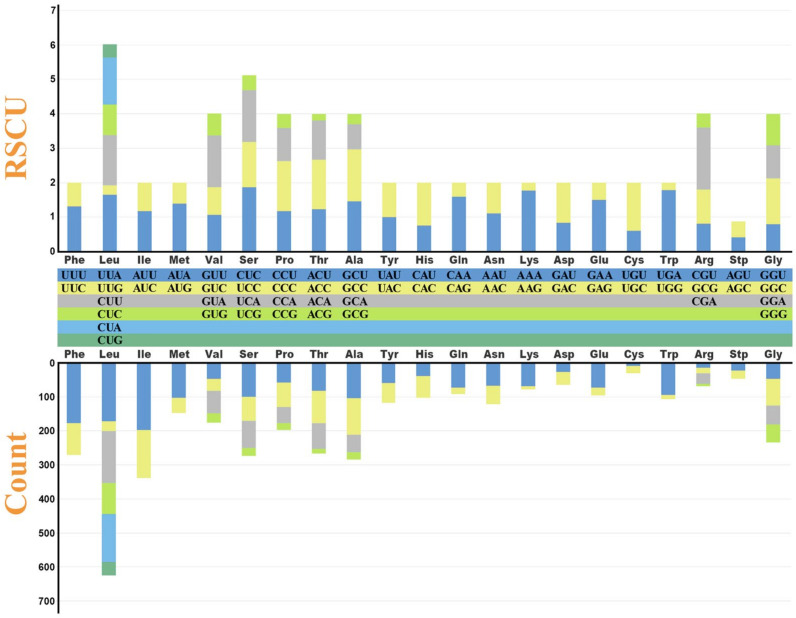
Relative synonymous codon usage (RSCU) in the mitogenome of *Oreolalax omeimontis*. The different colors in the column chart represent the codon families corresponding to the amino acids below and use consistent colors to represent the same codon families. The stop codon is excluded.

The relative synonymous codon usage (RSCU) values of the *O. omeimontis* mitogenome are shown in Figure 2 and Table S4. The results indicate that six codon families of UUU (177), UUA (172), CUU (153), CUA (141), AUU (198), and AUC (140) are most used in *O. omeimontis*, with more than 140 instances. In addition, six codon families (UUU, UUA, CUU, CUA, AUU, and AUC) are most used of all seven *Oreolalax* species, and the six least used codon families among the seven *Oreolalax* species were AAG, CGC, CGG, CGU, ACG, and UGG (Table S4). Obviously, overrepresentation of the Us (Ts) in frequently used codons indicates that the codon usage of PCGs contributed to both the A + T bias, and that Ts (Us) are the most abundant base of *Oreolalax* mitogenomes (Table 2). As for the codon RSCU values of seven *Oreolalax* species, *O. omeimontis*, *O. xiangchengensis*, *O. multipunctatus,* and *O. major* shared UCU (encoding serine) as their common codon with highest RSCU value, ranging from 2.17 (*O. major*) to 1.87 (*O. omeimontis*); while *O. rhodostigmatus*, *O. jingdongensis,* and *O. lichuanensis* had the highest RSCU value for CGA (encoding arginine) as their common codon with highest RSCU value, ranging from 2.17 (*O. major*) to 1.87 (*O. omeimontis*) (Table S4). Moreover, the coding codon with the lowest RSCU value of *O. omeimontis* was ACG (encoding threonine; with an RSCU value of 0.20), while that of *O. xiangchengensis* was CCG (proline; 0.14), *O. multipunctatus* was UCG (serine; 0.19), *O. major* was AAG (lysine; 0.10), *O. lichuanensis* was GCG (alanine; 0.20), whereas *O. rhodostigmatus* and *O. jingdongensis* had their lowest RSCU value for codon CGG (0.11 for *O. rhodostigmatus* and 0.16 for *O. jingdongensis*), which encodes arginine (Table S4).

### 3.3. Transfer RNA

**Figure 3:**
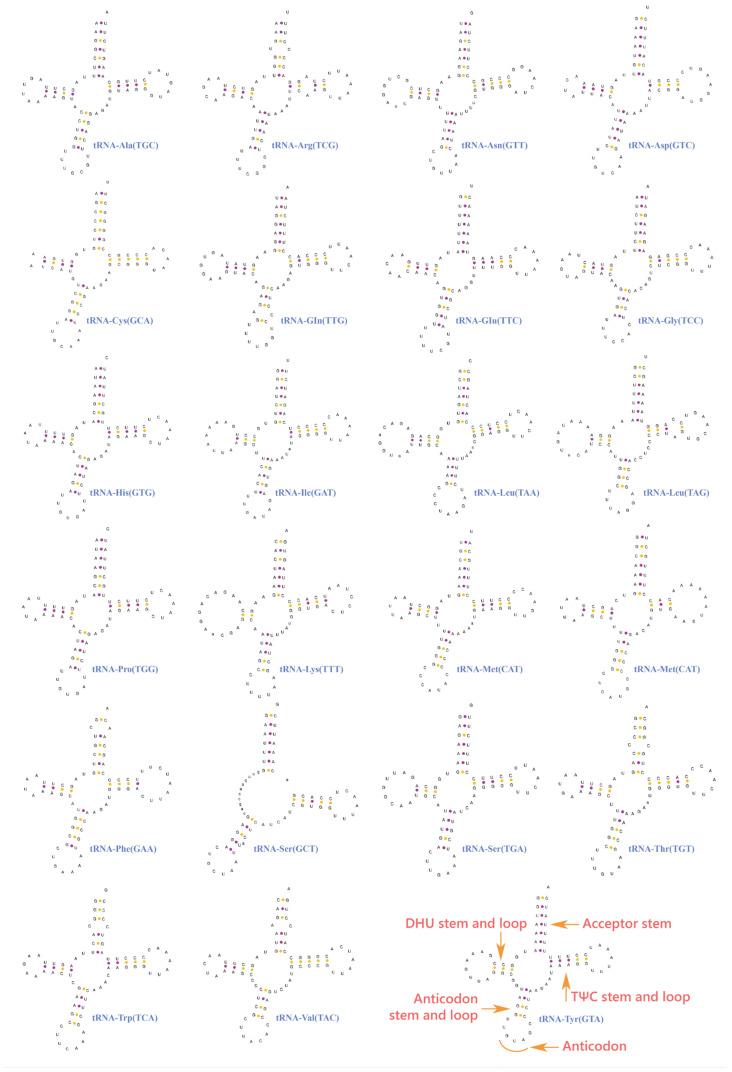
Putative secondary structures for 23 tRNA genes of *Oreolalax omeimontis*.

A total of 23 tRNA genes were found in the mitogenome of *O. omeimontis*, including an additional copy of *trnM*, which is consistent with other studies of *Oreolalax* species [13,14,15]. The tRNA genes ranged in size from 64 (*trnC*) to 75 bp (*trnL2*). Among them, eight tRNA genes were located on the L-strand (-) and the remaining 15 are located on the H-strand (+). Most of the tRNA genes were found to fold into the typical clover-leaf structure (Figure 3). We found a high degree of similarity between two *trnM* of *O. omeimontis* and between all the *trnM* form the other six *Oreolalax* species. In addition, the secondary structures of the two *trnM* sequences were predicted successfully. They had the same anticodon of CAU and could fold into the typical clover-leaf structure (Figure 3). This result indicates that neither of the two *trnM* sequences had degenerated, although a small number of non-complementary and U-G base pairs existed in the stem regions. Stem mismatches are common within mitochondrial tRNA genes and are repaired via post-transcriptional editing [46,47]. Furthermore, a lack of the dihydrouridine (DHU) in the arm of *trnS1* does not seem to affect its function, according to previous studies [48,49].

### 3.4. Ribosomal RNA and Control Region

For the *O. omeimontis*, *rrnS* was 935 bp long, which was located between *trnF* and *trnV*; *rrnL* was 1600 bp long and was present between *trnV* and *trnL2*. For rRNAs, the total A + T content was 58.2%, with a positive AT-skew (0.124) and a negative GC-skew (−0.105) (Table 2), indicating that the content of As is highest and the content of Gs is lowest. In contrast, *O. omeimontis* had the lowest A + T content (58.2%) among the *Oreolalax* species (Table 2). Consistent with the most metazoan mitogenomes (Table 1), *O. omeimontis* contains only one control region, which was located between the *trnW* and *trnF* genes with 1166 bp in size. The CR had the highest A + T content (63.9%) in the mitogenome, with both negative AT-skew (−0.095) and GC-skew (−0.291) (Table 2). Moreover, in all seven *Oreolalax* species, the CR had the highest A + T content in mitogenomes with both negative AT-skew and GC- skew, indicating that bias exists toward Ts and Cs in *Oreolalax* in CRs.

### 3.5. Phylogenetic Analysis

**Figure 4:**
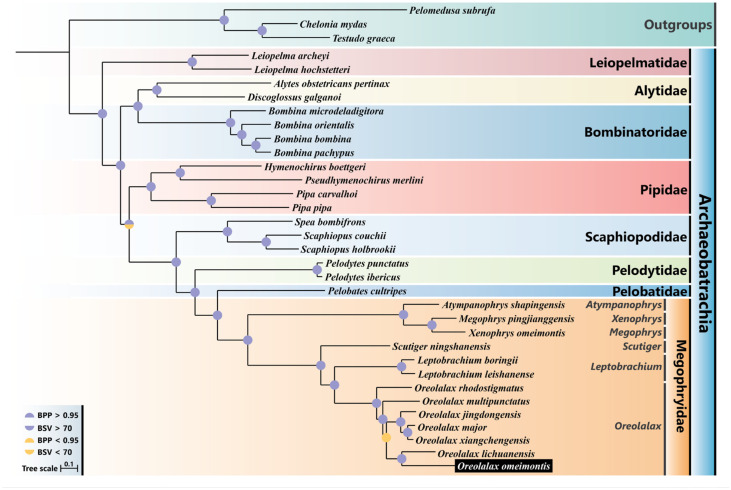
Phylogenetic tree of the Archaeobatrachia species based on the concatenated nucleotide sequences of 13 PCGs using maximum likelihood (ML) and Bayesian inference (BI) analysis. The semicircle on the upper of each node represents the Bayesian posterior probability (BPP), while the lower semicircle represents the bootstrap value (BSV).

Phylogenetic analyses were carried out based on the concatenated alignment of nucleotides sequences of 13 PCGs covering eight families and 31 species of Archaeobatrachia; three species were used as outgroups (Table S1). The final concatenated alignment of our mitogenome dataset for 34 species contained 11,478 nucleotide positions. All the topological structures from the Bayesian and ML analyses were consistent (Figure 4).

The present phylogenetic analysis of archaeobatrachians shows ((((((((*Megophryidae* + *Pelobatidae*) + *Pelodytidae*) + *Scaphiopodidae*)) + *Pipidae*)) + (*Bombinatoridae* + *Alytidae*)) + *Leiopelmatidae*) (Figure 4), which is consistent with previous studies [31,50]. The six genera of Megophryidae were clustered as (((*Oreolalax* + *Leptobrachium*) + *Scutiger*) + ((*Xenophrys* + *Megophrys*) + *Atympanophrys*)), which indicates that *Oreolalax* is sister group to *Leptobrachium*, and forms a branch with *Scutiger* (Figure 4). This result is consistent with Feng et al. [50] but inconsistent with Pyron et al. [25], who proposed that *Oreolalax* should be a sister group to *Scutiger* but not *Leptobrachium*. We believe that the incongruence between these two studies is that Feng et al. [50] used a longer sequence alignment with more genes (around 88,000 nt of aligned sequences from 95 nuclear protein-coding genes), while Pyron et al. [25], which predates Feng et al. [50] by six years, used only 12 genes (nine nuclear plus three mitochondrial genes in 12,712 nt) for phylogenetic purposes. As a result, both the previous study and ours were able to assemble more divergent loci to generate a more accurate phylogenetic structure.

Seven Oreolalax species clustered as ((((*O. omeimontis* + *O. lichuanensis*) + ((*O. major* + *O. xiangchengensis*) + *O. jingdongensis*)) + *O. multipunctatus*) + *O. rhodostigmatus*). Although there was low support for the sister group of (*O. omeimontis* + *O. lichuanensis*) + ((*O. major* + O. xiangchengensis) + O. jingdongensis) (BSV = 48; BPP = 0.73), the support values were high for other nodes in Oreolalax (BSV ≥ 70; BPP ≥ 95) (Figure 4). *O. rhodostigmatus* was placed at the basal position of the genus Oreolalax, which is consistent with previous studies that employed multi-markers [9,24,25]. *O. major* was a sister to *O. xiangchengensis* and *O. omeimontis* forms a sister relationship with *O. lichuanensis*, which was well supported by statistical data (bootstrap value, BSV = 100; Bayesian posterior probability, BPP = 1). Previous studies based on 13 mitochondrial PCGs [13,14,15] or partial nuclear and mitochondrial [24,25] data had the consistent view that *O. major* and *O. xiangchengensis* form a sister group. However, previous phylogenetic analyses based on multiple nuclear and mitochondrial gene fragments reconstructed *O. omeimontis* and *O. multipunctatus* as a sister branch [9,24,25], whereas our results based on 13 PCGs from the complete mitogenome were consistent with the phylogenetic results based on 21 morphological characters [23], i.e., *O. omeimontis* and *O. lichuanensis* were reconstructed as sister branch. Similar to the previous studies suggesting the use of longer sequences to obtain more accurate phylogenetic results [29], we believe that our study using 13 mitochondrial PCGs provided a more reliable phylogenetic relationship than research using a small number of gene fragments.

In general, our results demonstrated a different view to the studies with only a small number of gene fragments, as well as supported traditional approaches of using morphological data for phylogenic purposes, and also complemented phylogenetic analyses involving genus *Oreolalax*. Thus, more taxon sampling and more sequence data, including both nuclear and mitochondrial, as well as morphological markers, are required to obtain a more robust phylogenetic framework for *Oreolalax* taxa.

### 3.6. Gene Rearrangement

The organization of metazoan mitogenomes is usually conserved [3]. However, many cases of genome reorganization occur in closely related animals [19,51,52,53], so the mitogenome arrangements are thought to reflect phylogenetic relationships [51]. In addition, gene arrangements in mitogenomes are useful to describe the unusual genomic features and settle phylogenetic hypotheses for many Anura species [19,31]. In the Anura, most cases of gene rearrangement have been found in neobatrachians [53,54]. According to previous studies [17,18,19,31], most mitogenomes of archaeobatrachians have a common vertebrate gene order. However, since the publication of Anura mitogenomes, cases of gene rearrangement have been discovered in archaeobatrachians [13,14,15,55,56,57].

**Figure 5:**
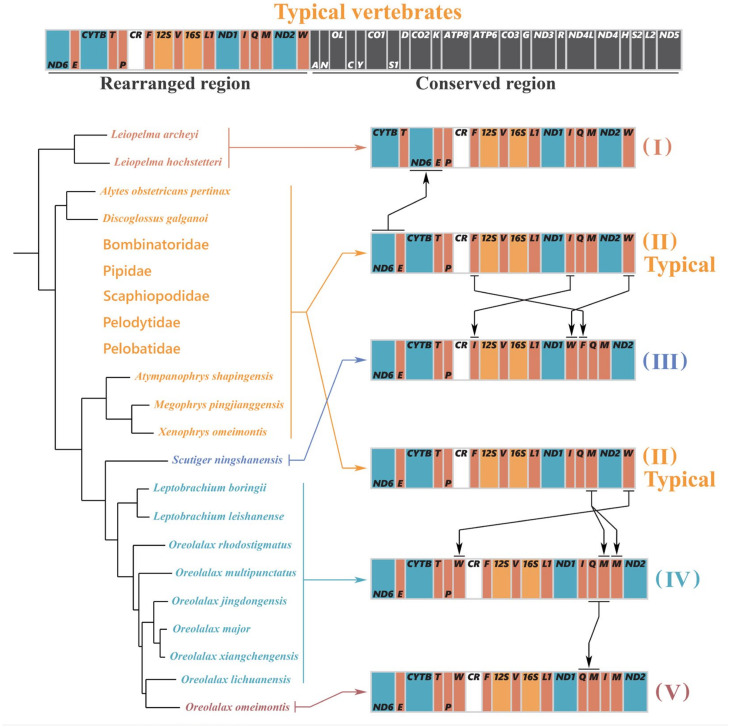
Diversity of gene rearrangements in *Archaeobatrachia* species. Lineages on which individual changes occurred were assigned using the parsimony criterion based on the phylogenetic framework (Figure 4). The mitogenomic features of each species on the tree are as follows: (I) translocation of *nad6* and *trnE*; (II) typical gene order; (III) translocation of *trnI*, *trnW,* and *trnF*; (IV) duplication of *trnM* and translocation of *trnW*; (V) translocation of *trnQ* and *trnM*.

In this study, the gene rearrangement events among 31 archaeobatrachians mitogenomes revealed five gene arrangement types (type I to type V); only the *O. omeimontis* mitogenome was recognized as type V (Figure 5). All species except *Leiopelmatidae* (type I) and partial *Megophryidae* species (types III, IV and V) have the typical (traditional) vertebrate mitogenome gene order (type II). Compared to typical mitogenome organization (type II), in the basal lineage of *Leiopelmatidae*, the two adjacent genes of *nad6* and *trnE* has been translocated from the canonical location (type II) between the *nad5* and *cob* genes to a region downstream of *trnT* (type I). Moreover, the translocation of *trnI*, *trnW,* and *trnF* seems to have occurred in *Scutiger ningshanensis* (type III), which is unique for other archaeobatrachians (Figure 5).

Additionally, duplication of *trnM* and translocation of *trnW* had both occurred in six *Oreolalax* species and two *Leptobrachium* species (type IV), and both *Oreolalax* and *Leptobrachium* were closely related in phylogenetic relationships, these gene rearrangement events may have occurred to the common ancestor of these two genera. Moreover, the translocations of *trnQ* and *trnM* in *O. omeimontis* seem to result from an independent event, as *O. omeimontis*’s arrangement (type V) is unique to other six *Oreolalax* species (Figure 5)

Gene rearrangements have been shown to occur independently in multiple lineages, which are relatively rare and random [19,57,58]. To explore whether there are more rearrangement events in the genus *Oreolalax* as well as *Archaeobatrachia* suborder, and to further investigate the mechanism of mitogenome rearrangement and evolution of Anura, further studies and more species of *Oreolalax* should be sequenced. Such follow-up studies could lead to new discoveries and insights into the evolution of mitogenome reorganization.

## 4. Conclusions

This study has presented the characterization of the complete mitogenome of *O. omeimontis*, which complements the molecular data of *Oreolalax* taxa. In addition, the gene arrangement of *O. omeimontis,* surprisingly, was revealed to be different from the other six published *Oreolalax* species. Furthermore, phylogenetic analyses were conducted for some Archaeobatrachia species and confirmed the phylogenic position of *O. omeimontis* as a sister species to *O. lichuanensis* by using 13 mitochondrial PCGs. In general, this study provides a novel insight into the mitochondrial gene arrangements and phylogenetic framework of the *Archaeobatrachia* suborder, and also facilitates future studies on the evolution of vertebrates. Meanwhile, further sampling from different taxonomic grades for mitogenomic and nuclear data will be helpful in understanding the phylogenetic and evolutionary implications among the species of *Archaeobatrachia*.

## Figures and Tables

**Table 1:** The mitogenome organization of *Oreolalax omeimontis*.

Features	Strand	Location	Size (bp)	Anti-codon	Start Codon	Stop Codon	IGS ^1^
*trnF*	+	1–68	68	GAA			0
*rrnS*	+	69–1003	935				0
*trnV*	+	1004–1072	69	TAC			0
*rrnL*	+	1073-2672	1600				0
*trnL2*	+	2673–2747	75	TAA			0
*nad1*	+	2748–3725	978		ATG	TAA	38
*trnQ*	–	3764–3834	71	TTG			21
*trnM*	+	3956–4023	68	CAT			163
*trnI*	+	4187–4258	72	GAT			107
*trnM*	+	4366–4435	72	CAT			1
*nad2*	+	4437–5480	1044		ATG	TAA	10
*trnA*	–	5491–5560	70	TGC			0
*trnN*	–	5561–5633	73	GTT			2
O_L_	+	5636–5662	27				−1
*trnC*	–	5662–5725	64	GCA			0
*trnY*	–	5726–5795	70	GTA			1
*cox1*	+	5797–7359	1563		GTG	AGG	−9
*trnS2*	–	7351–7421	71	TGA			4
*trnD*	+	7426–7493	68	GTC			0
*cox2*	+	7494-8175	682		ATG	T(AA)	0
*trnK*	+	8176–8249	74	TTT			0
*atp8*	+	8250–8417	168		ATG	TAA	−10
*atp6*	+	8408–9090	683		ATG	TA(A)	−1
*cox3*	+	9090–9874	785		ATG	TA(A)	−1
*trnG*	+	9874–9943	70	TCC			0
*nad3*	+	9944-10,286	343		ATG	T(AA)	0
*trnR*	+	10,287–10,355	69	TCG			0
*nad4l*	+	10,356–10,652	297		ATG	TAA	−7
*nad4*	+	10,646–12,024	1379		ATG	TA(A)	−1
*trnH*	+	12,024–12,092	69	GTG			0
*trnS1*	+	12,093–12,159	67	GCT			0
*trnL1*	+	12,160–12,231	72	TAG			6
*nad5*	+	12,238–14,058	1821		ATT	AGG	4
*nad6*	–	14,063–14,572	510		GTG	AGG	0
*trnE*	–	14,573–14,641	69	TTC			3
*cob*	+	14,645–15,785	1141		ATG	T(AA)	0
*trnT*	+	15,786–15,855	70	TGT			2
*trnP*	–	15,858–15,926	69	TGG			15
*trnW*	–	15,942–16,010	69	TCA			0
CR	+	16,011–17,266	1166				0

^1^ IGS = Intergenic Spacer.

**Table 2:** Base composition and skewness of the seven *Oreolalax* species.

Species	Complete Mitogenome			tRNAs			PCGs		
	A + T%	AT-Skew	GC-Skew	A + T%	AT-Skew	GC-Skew	A + T%	AT-Skew	GC-Skew
*O. omeimontis*	58.9	−0.063	−0.278	58.1	0.026	0.031	58.5	−0.145	−0.282
*O. lichuanensis*	60.2	−0.070	−0.248	58.5	0.022	0.055	59.8	−0.151	−0.249
*O. jingdongensis*	61.8	−0.058	−0.251	57.9	0.036	0.040	61.5	−0.138	−0.252
*O. xiangchengensis*	62.2	−0.061	−0.249	58.4	0.027	0.017	62.5	−0.142	−0.248
*O. rhodostigmatus*	60.4	−0.073	−0.258	57.8	0.017	0.047	59.8	−0.140	−0.254
*O. multipunctatus*	61.5	−0.073	−0.257	59.3	0.025	0.039	60.8	−0.145	−0.250
*O. major*	61.2	−0.059	−0.260	57.4	0.035	0.047	61.3	−0.132	−0.266
**Species**	**rRNAs**			**CR**					
	**A + T%**	**AT-Skew**	**GC-Skew**	**A + T%**	**AT-Skew**	**GC-Skew**			
*O. omeimontis*	58.2	0.124	−0.105	63.9	−0.095	−0.291			
*O. lichuanensis*	59.5	0.109	−0.101	62.6	−0.089	−0.212			
*O. jingdongensis*	59.9	0.129	−0.107	67.0	−0.084	−0.261			
*O. xiangchengensis*	60.3	0.121	−0.106	63.7	−0.068	−0.247			
*O. rhodostigmatus*	59.3	0.116	−0.115	65.4	−0.125	−0.246			
*O. multipunctatus*	60.0	0.107	−0.110	68.4	−0.099	−0.325			
*O. major*	59.5	0.126	−0.104	63.9	−0.121	−0.247			

## Data Availability

Data are contained within the article or Supplementary Materials.

## Supplementary Materials

The following supporting information can be downloaded at: https://www.mdpi.com/article/10.3390/genes13112089/s1, Table S1: List of the mitogenomes of species analyzed in this study and their GenBank accession numbers; Table S2: Best partitioning schemes selected by ModelFinder (13PCGS - ML) and PartitionFinder2 (13PCGS - BI), respectively; Table S3. Usage of start and stop codons in the mitogenomes of seven species of Oreolalax; Table S4. Condon number and RSCU of seven *Oreolalax* species mitochondrial PCGs. The asterisk in parentheses indicates the stop codon.
