# Supplementary material for: Complete Mitogenome of Oreolalax omeimontis Reveals Phylogenetic Status and Novel Gene Arrangement of Archaeobatrachia
Source: Genes (Basel). 2022 Nov 10;13(11):2089. doi: 10.3390/genes13112089 (PMC9691213; doi:10.3390/genes13112089)
Supplement: Supplementary file 1 [file genes-13-02089-s001.zip › Supplementary Tables.pdf]

Table S1. List of the mitogenomes of species analyzed in this study and their GenBank accession numbers.

Species	Family	Genus	GenBank No.
Alytes obstetricans pertinax	Alytidae	*Alytes*	AY585337
Discoglossus galganoi	Alytidae	*Discoglossus*	AY585339
Leiopelma archeyi	Leiopelmatidae	*Leiopelma*	HM142901
Leiopelma hochstetteri	Leiopelmatidae	*Leiopelma*	KJ792087
Bombina orientalis	Bombinatoridae	*Bombina*	AY585338
Bombina bombina	Bombinatoridae	*Bombina*	EU115993
Bombina pachypus	Bombinatoridae	*Bombina*	JX893179
Bombina microdeladigitora	Bombinatoridae	*Bombina*	JX893182
Pipa pipa	Pipidae	*Pipa*	GQ244477
Hymenochirus boettgeri	Pipidae	*Hymenochirus*	HM991331
Pipa carvalhoi	Pipidae	*Pipa*	HM991332
Pseudhymenochirus merlini	Pipidae	*Pseudhymenochirus*	HM991333
Scaphiopus couchii	Scaphiopodidae	*Scaphiopus*	JX564894
Spea bombifrons	Scaphiopodidae	*Spea*	JX564896
Scaphiopus holbrookii	Scaphiopodidae	*Scaphiopus*	KY962390
Pelodytes ibericus	Pelodytidae	*Pelodytes*	JX564882
Pelodytes punctatus	Pelodytidae	*Pelodytes*	JF703231
Pelobates cultripes	Pelobatidae	*Pelobates*	AJ871086
Atympanophrys shapingensis	Megophryidae	*Atympanophrys*	JX458090
Leptobrachium boringii	Megophryidae	*Leptobrachium*	KJ630505
Leptobrachium leishanense	Megophryidae	*Leptobrachium*	KU760082
Scutiger ningshanensis	Megophryidae	*Scutiger*	KX619450
Xenophrys omeimontis	Megophryidae	*Xenophrys*	KP728257
Megophrys pingjianggensis	Megophryidae	*Megophrys*	KT601071
Oreolalax lichuanensis	Megophryidae	*Oreolalax*	KU096847
Oreolalax major	Megophryidae	*Oreolalax*	KU127230
Oreolalax rhodostigmatus	Megophryidae	*Oreolalax*	MF770485
Oreolalax jingdongensis	Megophryidae	*Oreolalax*	MF953479
Oreolalax multipunctatus	Megophryidae	*Oreolalax*	MF966382
Oreolalax xiangchengensis	Megophryidae	*Oreolalax*	MH727696
Oreolalax omeimontis	Megophryidae	*Oreolalax*	OP722573
Pelomedusa subrufa	Pelomedusidae	*Pelomedusa*	AF039066
Chelonia mydas	Cheloniidae	*Chelonia*	AB012104
Testudo graeca	Testudinidae	*Testudo*	DQ080050

Table S2. Best partitioning schemes selected by ModelFinder (13PCGS - ML) and PartitionFinder2 (13PCGS - BI), respectively.

Data Matrix	Subset Partitions	Best Model
	1: *nad3*_ pos1, *atp6*_ pos1, *nad4*_ pos1	GTR+I+G
	2: *atp6*_ pos2, *nad1*_ pos2	GTR+I+G
	3: *nad3*_ pos3, *atp6*_ pos3	GTR +G
	4: *atp8*_ pos1	HKY+I+G
	5: *atp8*_ pos2	GTR+I+G
	6: *atp8*_ pos3	HKY+G
	7: *cox1*_ pos1	SYM +G
	8: *cox1*_ pos2	GTR+I+G
	9: *cox1*_ pos3	GTR+I+G
	10: *cox2*_ pos1	GTR+I+G
	11: *cox2*_ pos2	HKY+I+G
	12: *cox2*_ pos3	GTR+I+G
	13: *cox3*_ pos1	SYM +G
	14: *cox3*_ pos2, *cob*_ pos2	GTR+I+G
13PCGS - BI	15: *cox3*_ pos3	GTR+G
	16: *cob*_ pos1, *nad1*_ pos1	GTR+I+G
	17: *cob*_ pos3	GTR+G
	18: *nad1*_ pos3, *nad5*_ pos3, *nad4*_ pos3	GTR+I+G
	19: *nad2*_ pos1, *nad5*_ pos1	GTR+I+G
	20: *nad2*_ pos2	GTR+I+G
	21: *nad2*_ pos3	GTR+G
	22: *nad3*_ pos2, *nad4*_ pos2	GTR+I+G
	23: *nad4l*_ pos1	SYM+I+G
	24: *nad4l*_ pos2	GTR+G
	25: *nad4l*_ pos3	HKY +G
	26: *nad5*_ pos2	GTR+I+G
	27: *nad6*_ pos1	GTR+G
	28: *nad6*_ pos2	GTR+G
	29: *nad6*_ pos3	GTR+I+G

Table S2. *Cont.*

Data Matrix	Subset Partitions	Best Model
	1: *nad1*_ pos1, *cob*_ pos1	GTR+I+G
	2: *nad4*_ pos2, *nad1*_ pos2, *atp6*_ pos2	GTR+I+G
	3: *nad3*_ pos3, *nad1*_ pos3, *nad4l*_ pos3, *nad4*_ pos3, *atp6*_ pos3	GTR+I+G
	4: *nad2*_ pos1, *nad5*_ pos1	GTR+I+G
	5: *nad2*_ pos2, *atp8*_ pos2	GTR+G
	6: *nad2*_ pos3	GTR+G
	7: *cox1*_ pos1, *cox3*_ pos1	GTR+I+G
	8: *cox3*_ pos2, *cox1*_ pos2	GTR+I+G
	9: *cox1*_ pos3	GTR+G
	10: *cox2*_ pos1	GTR+I+G
	11: *cox2*_ pos2	GTR+G
13PCGS - ML	12: *atp8*_ pos3, *cox2*_ pos3, *cox3*_ pos3	GTR+G
	13: *atp8*_ pos1	GTR+G
	14: *atp6*_ pos1, *nad4*_ pos1	GTR+I+G
	15: *nad3*_ pos1, *nad4l*_ pos1	GTR+G
	16: *nad4l*_ pos2, *nad3*_ pos2	GTR +G
	17: *nad5*_ pos2	GTR+I+G
	18: *nad5*_ pos3	GTR+G
	19: *nad6*_ pos1	GTR+G
	20: *nad6*_ pos2	GTR+G
	21: *nad6*_ pos3	GTR+G
	22: *cob*_ pos2	GTR+I+G
	23: *cob*_ pos3	GTR+G

Table S3. Usage of start and stop codons in the mitogenomes of seven species of *Oreolalax*.

Species	***nad1***		***nad2***		***cox1***		***cox2***		***atp8***		***atp6***		***cox3***	
	**Start**	**Stop**	**Start**	**Stop**	**Start**	**Stop**	**Start**	**Stop**	**Start**	**Stop**	**Start**	**Stop**	**Start**	**Stop**
O. omeimontis	ATG	TAA	ATG	TAA	GTG	AGG	ATG	T(AA)	ATG	TAA	ATG	TA(A)	ATG	TA(A)
O. lichuanensis	ATG	TA(A)	ATG	TAA	GTG	AGA	ATG	T(AA)	ATG	TAA	ATG	TA(A)	ATA	TAA
O. jingdongensis	ATG	TA(A)	ATG	TAG	GTG	AGG	GTG	TAA	ATG	TAA	ATG	TA(A)	ATG	TA(A)
O. xiangchengensis	ATG	TA(A)	ATG	TAA	GTG	AGG	GTG	T(AA)	ATG	TAA	ATG	TA(A)	ATG	TA(A)
O. rhodostigmatus	ATG	TA(A)	ATG	TAA	GTG	AGA	ATG	T(AA)	GTG	TGA	ATG	TA(A)	ATG	TA(A)
O. multipunctatus	ATG	TA(A)	ATG	TAA	GTG	AGG	ATG	T(AA)	ATG	TAA	ATG	TA(A)	ATG	TA(A)
O. major	ATG	TA(A)	ATG	TAA	ATG	AGG	GTG	T(AA)	ATG	TAA	ATG	TA(A)	ATG	TA(A)

Species	***nad3***		***nad4l***		***nad4***		***nad5***		***nad6***		***Cob***	
	**Start**	**Stop**	**Start**	**Stop**	**Start**	**Stop**	**Start**	**Stop**	**Start**	**Stop**	**Start**	**Stop**
O. omeimontis	ATG	T(AA)	ATG	TAA	ATG	TA(A)	ATT	AGG	GTG	AGG	ATG	T(AA)
O. lichuanensis	ATT	T(AA)	ATG	TAA	ATG	TA(A)	GTG	AGA	GTG	AGG	ATG	T(AA)
O. jingdongensis	ATA	TAA	ATG	TAA	ATG	T(AA)	ATG	TAA	GTG	AGG	ATG	T(AA)
O. xiangchengensis	ATG	TA(A)	ATG	TAA	ATG	T(AA)	ATG	AGA	GTG	AGG	ATG	T(AA)
O. rhodostigmatus	ATA	TAA	ATG	TAA	ATG	T(AA)	ATA	AGG	ATG	AGG	ATG	T(AA)
O. multipunctatus	ATA	TAA	ATG	TA(A)	ATG	TA(A)	ATG	AGA	ATG	AGG	ATG	T(AA)
O. major	ATA	TAA	ATG	TAA	ATG	T(AA)	ATG	AGG	GTG	AGG	ATG	T(AA)

Table S4. Condon number and RSCU of seven *Oreolalax* species mitochondrial PCGs. The asterisk in parentheses indicates the stop codon.

Species	Codon	Count	RSCU	Codon	Count	RSCU	Codon	Count	RSCU	Codon	Count	RSCU
O. omeimontis	UUU(F)	177	1.31	UCU(S)	100	1.87	UAU(Y)	59	1	UGU(C)	9	0.6
	UUC(F)	94	0.69	UCC(S)	70	1.31	UAC(Y)	59	1	UGC(C)	21	1.4
	UUA(L)	172	1.65	UCA(S)	81	1.51	UAA (*)	4	2.29	UGA(W)	95	1.79
	UUG(L)	28	0.27	UCG(S)	23	0.43	UAG (*)	0	0	UGG(W)	11	0.21
	CUU(L)	153	1.47	CCU(P)	58	1.18	CAU(H)	39	0.76	CGU(R)	14	0.81
	CUC(L)	91	0.88	CCC(P)	71	1.44	CAC(H)	64	1.24	CGC(R)	17	0.99
	CUA(L)	141	1.36	CCA(P)	48	0.97	CAA(Q)	73	1.59	CGA(R)	31	1.8
	CUG(L)	39	0.38	CCG(P)	20	0.41	CAG(Q)	19	0.41	CGG(R)	7	0.41
	AUU(I)	198	1.17	ACU(T)	82	1.23	AAU(N)	67	1.11	AGU(S)	22	0.41
	AUC(I)	140	0.83	ACC(T)	95	1.43	AAC(N)	54	0.89	AGC(S)	25	0.47
	AUA(M)	102	1.39	ACA(T)	76	1.14	AAA(K)	69	1.77	AGA (*)	0	0
	AUG(M)	45	0.61	ACG(T)	13	0.2	AAG(K)	9	0.23	AGG (*)	3	1.71
	GUU(V)	47	1.07	GCU(A)	104	1.46	GAU(D)	27	0.84	GGU(G)	47	0.8
	GUC(V)	35	0.8	GCC(A)	107	1.51	GAC(D)	37	1.16	GGC(G)	78	1.33
	GUA(V)	66	1.5	GCA(A)	52	0.73	GAA(E)	72	1.5	GGA(G)	56	0.96
	GUG(V)	28	0.64	GCG(A)	21	0.3	GAG(E)	24	0.5	GGG(G)	53	0.91
O. lichuanensis	UUU(F)	179	1.31	UCU(S)	105	2.03	UAU(Y)	69	1.29	UGU(C)	18	1.24
	UUC(F)	95	0.69	UCC(S)	54	1.05	UAC(Y)	38	0.71	UGC(C)	11	0.76
	UUA(L)	185	1.77	UCA(S)	90	1.74	UAA (*)	4	2.67	UGA(W)	95	1.7
	UUG(L)	39	0.37	UCG(S)	12	0.23	UAG (*)	0	0	UGG(W)	17	0.3
	CUU(L)	148	1.42	CCU(P)	60	1.22	CAU(H)	41	0.83	CGU(R)	12	0.69
	CUC(L)	82	0.78	CCC(P)	67	1.37	CAC(H)	58	1.17	CGC(R)	15	0.86
	CUA(L)	133	1.27	CCA(P)	49	1	CAA(Q)	73	1.64	CGA(R)	37	2.11
	CUG(L)	40	0.38	CCG(P)	20	0.41	CAG(Q)	16	0.36	CGG(R)	6	0.34
	AUU(I)	223	1.35	ACU(T)	102	1.47	AAU(N)	67	1.06	AGU(S)	33	0.64
	AUC(I)	107	0.65	ACC(T)	76	1.09	AAC(N)	60	0.94	AGC(S)	16	0.31
	AUA(M)	102	1.4	ACA(T)	81	1.17	AAA(K)	65	1.6	AGA (*)	1	0.67
	AUG(M)	44	0.6	ACG(T)	19	0.27	AAG(K)	16	0.4	AGG (*)	1	0.67
	GUU(V)	52	1.05	GCU(A)	88	1.25	GAU(D)	30	0.9	GGU(G)	45	0.8
	GUC(V)	49	0.99	GCC(A)	116	1.65	GAC(D)	37	1.1	GGC(G)	64	1.14
	GUA(V)	63	1.27	GCA(A)	63	0.9	GAA(E)	71	1.56	GGA(G)	66	1.17
	GUG(V)	34	0.69	GCG(A)	14	0.2	GAG(E)	20	0.44	GGG(G)	50	0.89

Table S4. *Cont.*

Species	Codon	Count	RSCU	Codon	Count	RSCU	Codon	Count	RSCU	Codon	Count	RSCU
O. jingdongensis	UUU(F)	182	1.4	UCU(S)	96	1.89	UAU(Y)	71	1.25	UGU(C)	17	1.13
	UUC(F)	78	0.6	UCC(S)	49	0.96	UAC(Y)	43	0.75	UGC(C)	13	0.87
	UUA(L)	200	1.93	UCA(S)	100	1.97	UAA (*)	5	2.5	UGA(W)	98	1.77
	UUG(L)	26	0.25	UCG(S)	12	0.24	UAG (*)	1	0.5	UGG(W)	13	0.23
	CUU(L)	157	1.51	CCU(P)	79	1.6	CAU(H)	52	1.07	CGU(R)	21	1.14
	CUC(L)	60	0.58	CCC(P)	35	0.71	CAC(H)	45	0.93	CGC(R)	12	0.65
	CUA(L)	144	1.39	CCA(P)	66	1.34	CAA(Q)	77	1.69	CGA(R)	38	2.05
	CUG(L)	36	0.35	CCG(P)	17	0.35	CAG(Q)	14	0.31	CGG(R)	3	0.16
	AUU(I)	213	1.31	ACU(T)	110	1.57	AAU(N)	74	1.14	AGU(S)	25	0.49
	AUC(I)	113	0.69	ACC(T)	70	1	AAC(N)	56	0.86	AGC(S)	23	0.45
	AUA(M)	129	1.54	ACA(T)	85	1.21	AAA(K)	71	1.8	AGA (*)	0	0
	AUG(M)	39	0.46	ACG(T)	15	0.21	AAG(K)	8	0.2	AGG (*)	2	1
	GUU(V)	58	1.25	GCU(A)	117	1.64	GAU(D)	32	0.96	GGU(G)	55	0.98
	GUC(V)	42	0.9	GCC(A)	92	1.29	GAC(D)	35	1.04	GGC(G)	58	1.03
	GUA(V)	62	1.33	GCA(A)	60	0.84	GAA(E)	76	1.71	GGA(G)	71	1.26
	GUG(V)	24	0.52	GCG(A)	16	0.22	GAG(E)	13	0.29	GGG(G)	41	0.73
O. xiangchengensis	UUU(F)	185	1.43	UCU(S)	106	2.07	UAU(Y)	72	1.25	UGU(C)	17	1.17
	UUC(F)	74	0.57	UCC(S)	46	0.9	UAC(Y)	43	0.75	UGC(C)	12	0.83
	UUA(L)	209	2.04	UCA(S)	102	1.99	UAA (*)	3	2	UGA(W)	100	1.8
	UUG(L)	23	0.22	UCG(S)	7	0.14	UAG (*)	0	0	UGG(W)	11	0.2
	CUU(L)	171	1.67	CCU(P)	85	1.74	CAU(H)	49	0.99	CGU(R)	18	1
	CUC(L)	51	0.5	CCC(P)	29	0.59	CAC(H)	50	1.01	CGC(R)	15	0.83
	CUA(L)	135	1.31	CCA(P)	74	1.52	CAA(Q)	80	1.76	CGA(R)	31	1.72
	CUG(L)	27	0.26	CCG(P)	7	0.14	CAG(Q)	11	0.24	CGG(R)	8	0.44
	AUU(I)	240	1.49	ACU(T)	111	1.55	AAU(N)	80	1.2	AGU(S)	25	0.49
	AUC(I)	82	0.51	ACC(T)	76	1.06	AAC(N)	53	0.8	AGC(S)	21	0.41
	AUA(M)	130	1.51	ACA(T)	88	1.23	AAA(K)	73	1.83	AGA (*)	1	0.67
	AUG(M)	42	0.49	ACG(T)	11	0.15	AAG(K)	7	0.18	AGG (*)	2	1.33
	GUU(V)	69	1.43	GCU(A)	113	1.63	GAU(D)	36	1.11	GGU(G)	52	0.92
	GUC(V)	33	0.68	GCC(A)	92	1.32	GAC(D)	29	0.89	GGC(G)	55	0.98
	GUA(V)	70	1.45	GCA(A)	59	0.85	GAA(E)	73	1.64	GGA(G)	73	1.3
	GUG(V)	21	0.44	GCG(A)	14	0.2	GAG(E)	16	0.36	GGG(G)	45	0.8

Table S4. *Cont.*

Species	Codon	Count	RSCU	Codon	Count	RSCU	Codon	Count	RSCU	Codon	Count	RSCU
O. rhodostigmatus	UUU(F)	178	1.37	UCU(S)	91	1.8	UAU(Y)	60	1.1	UGU(C)	17	1.06
	UUC(F)	81	0.63	UCC(S)	58	1.14	UAC(Y)	49	0.9	UGC(C)	15	0.94
	UUA(L)	184	1.75	UCA(S)	86	1.7	UAA (*)	3	2	UGA(W)	90	1.59
	UUG(L)	33	0.31	UCG(S)	19	0.38	UAG (*)	0	0	UGG(W)	23	0.41
	CUU(L)	168	1.6	CCU(P)	70	1.39	CAU(H)	45	0.9	CGU(R)	13	0.73
	CUC(L)	73	0.7	CCC(P)	62	1.23	CAC(H)	55	1.1	CGC(R)	12	0.68
	CUA(L)	138	1.31	CCA(P)	59	1.17	CAA(Q)	76	1.63	CGA(R)	44	2.48
	CUG(L)	34	0.32	CCG(P)	11	0.22	CAG(Q)	17	0.37	CGG(R)	2	0.11
	AUU(I)	214	1.37	ACU(T)	92	1.32	AAU(N)	69	1.05	AGU(S)	22	0.43
	AUC(I)	99	0.63	ACC(T)	83	1.19	AAC(N)	62	0.95	AGC(S)	28	0.55
	AUA(M)	124	1.49	ACA(T)	90	1.29	AAA(K)	69	1.73	AGA (*)	1	0.67
	AUG(M)	42	0.51	ACG(T)	14	0.2	AAG(K)	11	0.28	AGG (*)	2	1.33
	GUU(V)	56	1.16	GCU(A)	109	1.55	GAU(D)	30	0.95	GGU(G)	47	0.83
	GUC(V)	49	1.02	GCC(A)	102	1.45	GAC(D)	33	1.05	GGC(G)	64	1.13
	GUA(V)	56	1.16	GCA(A)	52	0.74	GAA(E)	71	1.56	GGA(G)	61	1.07
	GUG(V)	32	0.66	GCG(A)	19	0.27	GAG(E)	20	0.44	GGG(G)	55	0.97
O. multipunctatus	UUU(F)	162	1.27	UCU(S)	105	2.01	UAU(Y)	70	1.21	UGU(C)	21	1.31
	UUC(F)	93	0.73	UCC(S)	54	1.04	UAC(Y)	46	0.79	UGC(C)	11	0.69
	UUA(L)	203	1.93	UCA(S)	94	1.8	UAA (*)	2	1.6	UGA(W)	98	1.77
	UUG(L)	35	0.33	UCG(S)	10	0.19	UAG (*)	0	0	UGG(W)	13	0.23
	CUU(L)	159	1.51	CCU(P)	77	1.56	CAU(H)	50	1.03	CGU(R)	13	0.73
	CUC(L)	85	0.81	CCC(P)	53	1.08	CAC(H)	47	0.97	CGC(R)	15	0.85
	CUA(L)	118	1.12	CCA(P)	56	1.14	CAA(Q)	82	1.74	CGA(R)	35	1.97
	CUG(L)	30	0.29	CCG(P)	11	0.22	CAG(Q)	12	0.26	CGG(R)	8	0.45
	AUU(I)	224	1.42	ACU(T)	106	1.5	AAU(N)	70	1.08	AGU(S)	25	0.48
	AUC(I)	91	0.58	ACC(T)	77	1.09	AAC(N)	60	0.92	AGC(S)	25	0.48
	AUA(M)	113	1.53	ACA(T)	81	1.15	AAA(K)	75	1.81	AGA (*)	1	0.8
	AUG(M)	35	0.47	ACG(T)	18	0.26	AAG(K)	8	0.19	AGG (*)	2	1.6
	GUU(V)	63	1.28	GCU(A)	112	1.58	GAU(D)	36	1.11	GGU(G)	48	0.85
	GUC(V)	32	0.65	GCC(A)	96	1.36	GAC(D)	29	0.89	GGC(G)	61	1.07
	GUA(V)	71	1.44	GCA(A)	54	0.76	GAA(E)	75	1.63	GGA(G)	73	1.29
	GUG(V)	31	0.63	GCG(A)	21	0.3	GAG(E)	17	0.37	GGG(G)	45	0.79

Table S4. *Cont.*

Species	Codon	Count	RSCU	Codon	Count	RSCU	Codon	Count	RSCU	Codon	Count	RSCU
O. major	UUU(F)	162	1.25	UCU(S)	110	2.17	UAU(Y)	69	1.19	UGU(C)	14	0.97
	UUC(F)	97	0.75	UCC(S)	46	0.91	UAC(Y)	47	0.81	UGC(C)	15	1.03
	UUA(L)	201	1.93	UCA(S)	87	1.72	UAA (*)	4	2.29	UGA(W)	97	1.75
	UUG(L)	26	0.25	UCG(S)	14	0.28	UAG (*)	0	0	UGG(W)	14	0.25
	CUU(L)	171	1.64	CCU(P)	78	1.6	CAU(H)	48	0.99	CGU(R)	18	0.99
	CUC(L)	58	0.56	CCC(P)	40	0.82	CAC(H)	49	1.01	CGC(R)	15	0.82
	CUA(L)	141	1.35	CCA(P)	65	1.33	CAA(Q)	83	1.8	CGA(R)	35	1.92
	CUG(L)	28	0.27	CCG(P)	12	0.25	CAG(Q)	9	0.2	CGG(R)	5	0.27
	AUU(I)	207	1.31	ACU(T)	96	1.32	AAU(N)	78	1.17	AGU(S)	26	0.51
	AUC(I)	109	0.69	ACC(T)	93	1.28	AAC(N)	55	0.83	AGC(S)	21	0.41
	AUA(M)	129	1.54	ACA(T)	93	1.28	AAA(K)	76	1.9	AGA (*)	0	0
	AUG(M)	38	0.46	ACG(T)	8	0.11	AAG(K)	4	0.1	AGG (*)	3	1.71
	GUU(V)	68	1.4	GCU(A)	102	1.48	GAU(D)	33	1.03	GGU(G)	51	0.91
	GUC(V)	40	0.82	GCC(A)	99	1.43	GAC(D)	31	0.97	GGC(G)	62	1.1
	GUA(V)	65	1.34	GCA(A)	62	0.9	GAA(E)	76	1.67	GGA(G)	66	1.17
	GUG(V)	21	0.43	GCG(A)	13	0.19	GAG(E)	15	0.33	GGG(G)	46	0.82
